# Effects of cumulus cell removal time during in vitro fertilization on embryo quality and pregnancy outcomes: a prospective randomized sibling-oocyte study

**DOI:** 10.1186/s12958-016-0151-3

**Published:** 2016-04-12

**Authors:** Na Guo, Fei Yang, Qun Liu, Xinling Ren, Hua Zhao, Yufeng Li, Jihui Ai

**Affiliations:** Department of Reproductive Medicine Center, Tongji Hospital, Tongji Medical College, Huazhong University of Science and Technology, 1095 Jiefang Avenue, Qiaokou District, Wuhan, 430030 China

**Keywords:** Sibling oocyte, Cumulus cell, In vitro fertilization

## Abstract

**Background:**

To investigate whether cumulus cell removal after a 3 h co-incubation of gametes can affect the outcomes of in vitro fertilization.

**Methods:**

A prospective randomized sibling-oocyte study was performed in which sibling oocytes obtained from each patient were randomly assigned to either a 3 h or 20 h group (cumulus cells removed at 3 h or 20 h after insemination, respectively). Same origin embryos (either 3 h or 20 h) were transferred. The study participants were patients < 38 years old and with infertility due to tubal factors. The study outcomes included fertilization, embryo quality, and birth status.

**Results:**

The study enrolled 172 patients, from whom 2275 oocytes were retrieved (1139 oocytes for the 3 h group and 1136 oocytes for the 20 h group). A total of 134 patients received embryo transfers (74 patients in the 3 h group and 60 patients in the 20 h group), and there were 54 live births (32 in the 3 h group and 22 in the 20 h group). When compared with patients in the 20 h group, patients in the 3 h group produced a larger number of optimal quality embryos, but had higher rates of polyspermy and low birth weight newborns. The two groups showed no differences in their rates of normal fertilization, pregnancy, and live birth.

**Conclusions:**

When compared with results obtained using a traditional cumulus cell removal protocol, early cumulus cell removal has both advantages and disadvantages. Further studies, and especially long-term outcome studies on newborns, need to be performed.

**Trial registration:**

Current controlled trial ChiCTR-OOC-15006878

## Background

Infertility can produce high levels of stress and have a negative impact on both individuals and society. Assisted reproductive technology, which includes several types of medical treatments, can improve the chance of pregnancy and a live birth. However, even with in vitro fertilization (IVF), which has the highest success rate among all types of assisted reproductive technology, only ~ 50 % of women achieve pregnancy [1]. Therefore, research on methods to improve the IVF process and its pregnancy outcomes is of great importance.

One major cause for failed IVF is failure of the sperm to fertilize the oocyte [2]. A rescue procedure, such as intracytoplasmic sperm injection (ICSI), can help improve the oocyte fertilization rate. Additionally, it was previously shown that early ICSI can increase pregnancy rates and lead to better clinical outcomes [3]. In order to recognize unfertilized oocytes and perform ICSI early on, oocytes have to be examined for the absence of a second polar body, and early cumulus cell removal can facilitate this examination [4]. Furthermore, early cumulus cell removal can also reduce the required co-incubation time between sperm and oocytes, and help eliminate degradation products from granulosa cells. These effects of early cumulus cell removal can reduce the potential for oocytes to be damaged by reactive oxygen species [5, 6]. However, previous studies which investigated the outcomes of early cumulus cell removal have shown conflicting results, with some studies reporting no change in pregnancy rates, and other studies reporting a decreased number of available embryos [7–9]. Such results suggest that further studies are needed to understand the effects of early cumulus cell removal during IVF on pregnancy outcomes.

We performed a prospective randomized sibling-oocytes study comparing the effects of cumulus cell removal performed at different time points on pregnancy outcomes.

## Methods

### Study design and patient selection

This prospective randomized clinical study was performed at the Reproductive Medicine Center of Tongji Hospital, Huazhong University of Science and Technology in China, between May 2012 and August 2013. The study protocol was approved by the hospital’s Institutional Review Board, and registered with the Chinese Clinical Trial Registry (registration number ChiCTR-OOC-15006878). Patients undergoing conventional IVF treatment were invited to participate in this study, and those who enrolled provided their signed written informed consent. The study inclusion criteria were an age < 38 years old and infertility due to tubal factors.

### Ovarian stimulation

All patients received a long protocol treatment of down-regulation with a gonadotrophin releasing hormone (GnRH) agonist (Decapeptyl, Ferring, Kiel, Germany; Diphereline, Ipsen, Paris, France) which was administrated daily starting on day 21 of the previous menstrual cycle. Individually adapted doses of follicle-stimulating hormone and/or human menopausal gonadotropins were injected after pituitary desensitization was confirmed. A single 10,000 unit dose of human chorionic gonadotropin was administrated when at least three ovary follicles reached a mean diameter of 17–18 mm; at which point, follicular aspiration was performed 36 h later.

### Sperm preparation

All semen samples were collected via masturbation following 3–7 days of sexual abstinence, and were allowed to liquefy for at least 30 min at 37 °C prior to the swim-up purification process. Sperm concentration, motility, and morphology were evaluated under a light microscope based on criteria established by the World Health Organization (WHO, 1992).

### In vitro insemination

Follicles were punctured using a double lumen oocyte harvesting needle. Each follicle was individually punctured, and each oocyte was cultured in one droplet. The first oocyte obtained from each patient was randomly assigned to either a cumulus cell 3 h removal group (3 h) or a standard 20 h removal group (20 h) on retrieval day (day 0). Subsequent oocytes obtained from the same patient were also assigned to either a 3 h or 20 h group. Following retrieval, the oocytes were incubated for 3 h in a single droplet containing 20,000-30,000 motile sperm.

### Cumulus cell removal

The cumulus cells and oocytes in the 3 h group were incubated with sperm for 3 h. Following incubation, the oocytes were rinsed and gently washed; after which, the cumulus cells were mechanically removed using a pipette with an inner diameter slightly smaller than that of the oocytes. After most of the cumulus cells were removed, the oocytes were transferred and cultured in a fresh medium. Fertilized oocytes were checked for the presence of a second polar body, and those without a second polar body were subjected to rescue ICSI. Oocytes in the 20 h group were inseminated for 3 h, and then transferred from the insemination medium to fresh medium with their surrounding cumulus cells left intact during transfer. On day 1, which was ~ 20 h after the oocyte retrievals, the cumulus cells were removed to allow an assessment of fertilization. Because early rescue ICSI is usually performed at 3 h after insemination, and cumulus cell removal is usually performed at 20 h [4, 8, 9], we chose 3 h and 20 h, respectively, as our early and late time points for cumulus cell removal.

### Embryo transfer

Embryos were transferred on day 3 after oocyte retrieval. Each patient received two transferred embryos, and patients received only same origin embryos (either 3 h or 20 h). Progesterone injections were initiated according to the conventional IVF treatment protocol. All patients were closely monitored for clinical pregnancy and birth.

### Outcome measurements

Normal fertilization was defined as the presence of two clearly visible pronuclei, together with two individualized or fragmented polar bodies at 20 h after IVF. An optimal embryo on day 3 contained ≥ 6 equal sized blastomeres, and showed a ≤ 10 % degree of fragmentation rate [10, 11]. Gardner’s scoring system was used to evaluate blastocysts. An optimal blastocyst was defined as a blastocoel fully filled embryo which displayed a tightly packed inner cell mass, and a trophectoderm with numerous cells which formed a cohesive epithelium on day 5 or 6. Embryos which reached the blastocyst stage (minimum expansion: early blastocyst; inner cell mass and trophectoderm layer: score A or B) were cryopreserved on day 5 or day 6. Clinical pregnancy was defined as the presence of a gestational sac that displayed a fetal heartbeat during ultrasound screening. Implantation rate was defined as the number of gestational sacs observed, divided by the number of embryos transferred. Premature delivery was defined as the baby being born at a gestational age of < 37 weeks. Birth weight was recorded in kilograms, and low birth weight was defined as < 2500 g.

### Statistical analysis

Continuous data are presented as the mean ± standard deviation, and ordinal data are presented as proportions. The data were analyzed by either the student’s t-test or chi-squared test using IBM SPSS Statistics for Windows Version 19.0 (IBM Corp., Armonk, NY). A *P*-value < 0.05 was considered statistically significant.

## Results

**Fig. 1:**
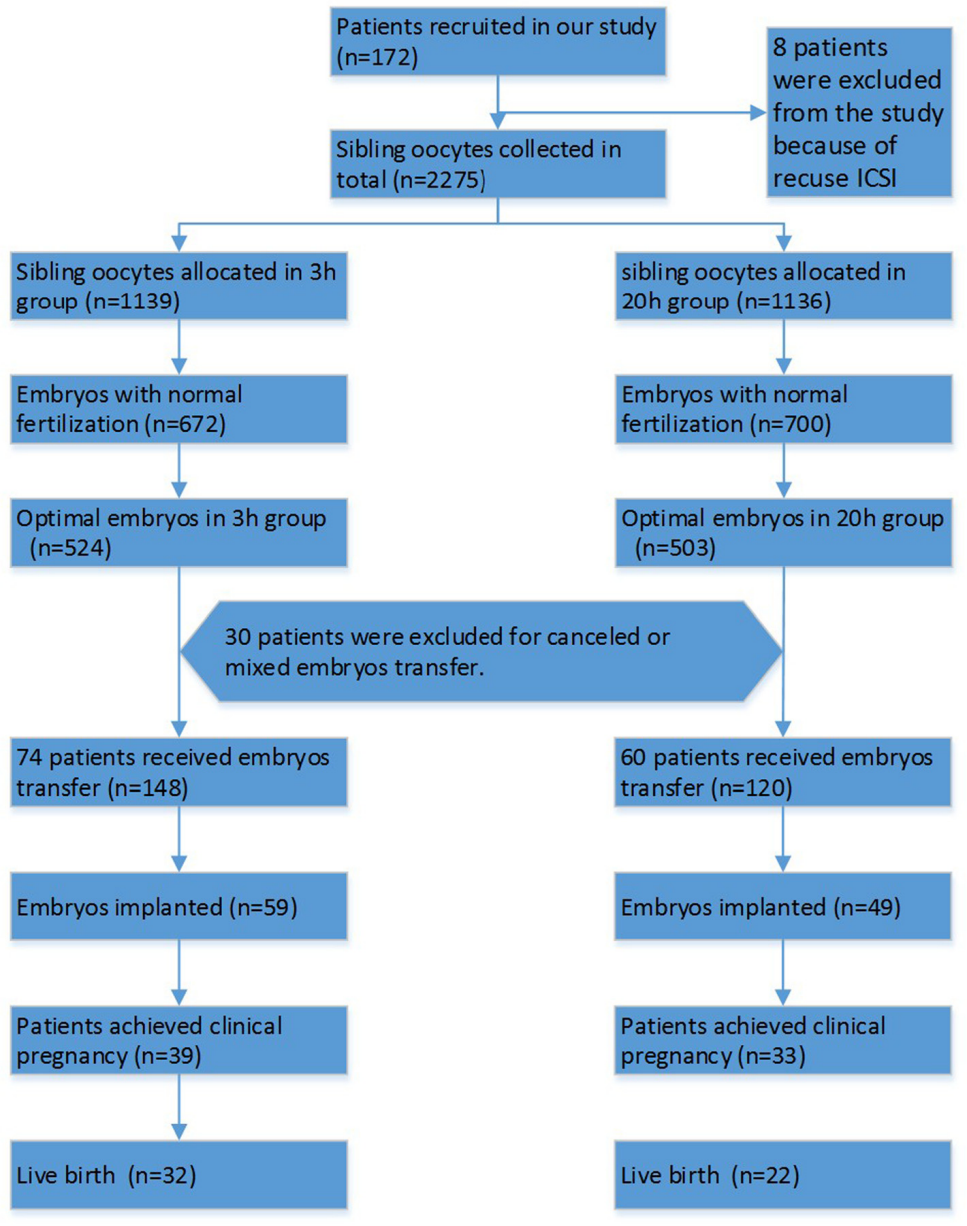
CONSORT flow diagram

The study recruited 172 patients; among whom, 134 patients were analyzed (Fig. 1, 74 patients in the 3 h group and 60 patients in the 20 h group). The mean age of the patients was 29.7 years (30.3 ± 3.7 years in the 3 h group and 29.3 ± 2.7 years in the 20 h group). The numbers of treatment cycles in the 3 h and 20 h groups were 1.0 ± 0.2 and 1.1 ± 0.4, respectively. Thirty patients were excluded due to either cancelled cycles (3 patients) or mixed embryo transfers (27 patients). Eight patients were not included in the statistical analysis because they received early rescue ICSI.

The fertilization, embryo, and pregnancy outcomes are shown in Tables 1, 2, and 3. When compared with the 20 h group, the 3 h group had a significantly higher polyspermy rate and percentage of optimal embryos. An analysis on obstetric outcomes showed that low birth weight infants were more common in the 3 h group (Table 4).

**Table 1:** Comparison of fertilization characteristics between two groups

	3 h group	20 h group	*P*
Oocytes obtained, (n)	1139	1136	
Metaphase II oocytes, n (%)	1066 (93.6)	1040(91.5)	0.06
Normal fertilization (2PN), n (%)	672 (59.0)	700 (61.6)	0.20
Polyspermy rate (>2PN), n (%)	226 (19.8)	171(15.1)	0.01

**Table 2:** Comparison of embryo characteristics between two groups

	3 h group	20 h group	*P*
Cleavage rate, n (%)	988(86.7)	969(85.3)	0.32
Available embryo rate, n (%)	705(61.9)	718(63.2)	0.52
Optimal embryo rate, n (%)	524(78)	503(71.9)	0.01
Blastocyst formation rate, n (%)	323(63.7)	342(61.5)	0.46
Optimal blastocyte rate, n (%)	138(42.7)	140(40.9)	0.64
Frozen blastocyst rate, n (%)	211(65.3)	225(65.8)	0.90

**Table 3:** Comparison of pregnancy outcomes between two groups

	3 h group	20 h group	*P*
Patients recruited, (n)	74	60	
Embryos transfer, (n)	148	120	
Mean number of embryos transferred, (n)	2.0	2.0	
Implantation rate, n (%)	59(39.9)	49(40.8)	0.87
Clinical pregnancy rate, n (%)	39(52.7)	33(55)	0.07
Ongoing pregnancy rate, n (%)	35(47.3)	28(46.7)	0.90
Live birth rate, n (%)	32(43.2)	22(36.7)	0.40
Twins rate, n (%)	16(50)	7(31.8)	0.18
Premature delivery rate, n (%)	11(14.9)	5(8.3)	0.20

**Table 4:** Comparison of obstetric outcomes between two groups

	3 h group	20 h group	*P*
Live birth fetuses, (n)	48	31	
Premature delivery fetus, (n)	21	8	
Gender ratio,(male/female)	1.3	0.9	0.50
Birth weight(Kg), mean ± SD	2.8 ± 0.5	2.9 ± 0.4	0.20
Low birth weight rate, n(%)	16(33.3)	4(12.9)	0.04

## Discussion

While early cumulus cell removal can facilitate early rescue ICSI and might improve pregnancy outcomes [3, 12], several previous studies have reported inconsistent results regarding outcomes after early cumulus cell removal. Some studies reported that early cumulus cell removal resulted in fertilization and clinical pregnancy rates similar to those obtained when performing a standard removal at 20 h post-insemination [8]. Another study showed that early cumulus removal after 4 h of insemination was associated with low numbers of available embryos [7]. Our current prospective randomized sibling-oocyte study demonstrated that early cumulus cell removal resulted in larger numbers of optimal embryos, but also a higher rate of polyspermy and a larger percentage of low birth weight infants. These types of results have not been previously reported.

Previous studies have demonstrated that co-culture of human oocytes and cumulus cells can improve embryo morphology and blastocyst formation [13, 14]. This might be due to bi-directional communications between oocytes and cumulus cells [15]. Cumulus cells provide oocytes with a local source of glycosaminoglycans, steroid hormones, nutrients, and other factors during stages of oocyte nuclear and cytoplasmic maturation, fertilization, and development [16]. However, our study showed that early cumulus cell removal can increase the quality of embryos. This is probably because cumulus cell removal reduces the levels of toxic metabolic products (e.g., active oxygen species, E2, and progesterone) produced by sperm and cumulus cells, and thus the adverse effects of these products on the vitality and quality of embryos [5, 6]. Some researchers have demonstrated a significant negative relationship between the incidence of cumulus cell apoptosis and the “good-embryo” rate [6]. Cumulus cells may have both beneficial and adverse effects on oocytes, and the exact nature of these effects require further investigation.

Our study showed a higher polyspermy rate among patients in the 3 h group. Cumulus cells are more difficult to remove at 2–4 h after insemination when compared with their removal at 20 h. However, at early times after insemination, the oocytes are also more vulnerable, due to their active spindles and microtubules [7, 8]. This can result in their greater susceptibility to being damaged by the additional mechanical stress created when a denuding pipette is used remove cumulus cells. Moreover, repeated mechanical stimulation can also have detrimental effects on the integrity of an oocyte’s zona pellucid, and thus reduce its defense against polyspermy. Polyspermy might also be caused by unstable culture conditions or an excessive number of sperm in the culture medium, which may be directly or indirectly related to early cumulus cell removal. All of these factors may have contributed to the increased polyspermy rate found in the current study’s 3 h group. However, our study did not find a significant difference between the rates of normal fertilization in the 3 h and 20 h groups, suggesting that normal fertilization was not affected by early cumulus cell removal. We also observed a high rate of low birth weight newborns in the 3 h group. Whether these low birth weight newborns were a result of mechanical stimulation during cumulus cell removal remains unknown, and requires further study.

Our current study showed that when compared with the 20 h group, the 3 h group had poorer obstetric and prenatal outcomes. Also, while the differences were not statistically significant, patients in the 3 h group had higher premature deliver and twin delivery rates, as well as a higher chance of having a low birth weight newborn. One possible explanation for these findings might be injuries that occurred due to mechanical stimulation during the stripping of cumulus cells from oocytes. Also, inappropriate repeated mechanical stimulation can cause epigenetic-based changes resulting from DNA methylation and gene differential expression; both of which are related to the incidence of low birth weight newborns [17, 18]. Additionally, it has been shown that removal of cumulus cells by high-speed vortexing can affect the relative locations of chromosome spindles and the first polar body [19]. Such changes might result in poor fetal development, twin pregnancy or early delivery. Other alterations created by cumulus cell retrieval, such as imprinted genes, subtle epigenetic alterations, and epigenetic reprogramming, might also contribute to a higher incidence of low birth weight newborns [20].

Our study has some limitations that should be mentioned. First, the study was conducted at a single hospital. Therefore, our results may not be applicable to other hospitals, because different hospitals may use different ovarian stimulation and IVF protocols. Second, our study population only included patients with infertility due to tubal factors, and not other causes. Similar studies should be performed at other hospitals, and with patients having other causes for infertility.

## Conclusions

In conclusion, our prospective randomized sibling-oocyte study showed that early cumulus cell removal at 3 h after insemination resulted in embryos of greater quality, but also higher rates of polyspermy and low birth weight infants. These findings have never been previously reported. Further studies are required to investigate the advantages and disadvantages of early cumulus cell removal. A study which includes the long-term follow-up of newborns as a means to evaluate the effects of cumulus cell removal should also be performed.

## Consent to publish

The study participants provided their consent to publish this article.

## Ethical approval

All procedures involving human participants were performed in accordance with ethical standards developed by an institutional and/or national research committee, and were in compliance with the 1964 Helsinki declaration and its later amendments, or comparable ethical standards.
